# Gene expression variation in Down's syndrome mice allows prioritization of candidate genes

**DOI:** 10.1186/gb-2007-8-5-r91

**Published:** 2007-05-25

**Authors:** Marc Sultan, Ilaria Piccini, Daniela Balzereit, Ralf Herwig, Nidhi G Saran, Hans Lehrach, Roger H Reeves, Marie-Laure Yaspo

**Affiliations:** 1 Max Planck Institute for Molecular Genetics, Ihnestr.63/73, 14195, Berlin, Germany; 2 Department of Physiology, Johns Hopkins University School of Medicine, 725 N. Wolfe St., Baltimore, Maryland 21205, USA; 3 McKusick-Nathans Institute of Genetic Medicine, 733 Nth. Broadway, Johns Hopkins University School of Medicine, Baltimore, Maryland 21205, USA

## Abstract

**Background:**

Down's syndrome (DS), or trisomy 21, is a complex developmental disorder that exhibits many clinical signs that vary in occurrence and severity among patients. The molecular mechanisms responsible for DS have thus far remained elusive. We argue here that normal variation in gene expression in the population contributes to the heterogeneous clinical picture of DS, and we estimated the amplitude of this variation in 50 mouse orthologs of chromosome 21 genes in brain regions of Ts65Dn (a mouse model of DS). We analyzed the RNAs of eight Ts65Dn and eight euploid mice by real-time polymerase chain reaction.

**Results:**

In pooled RNAs, we confirmed that trisomic/euploid gene expression ratios were close to 1.5. However, we observed that inter-individual gene expression levels spanned a broad range of values. We identified three categories of genes: genes with expression levels consistently higher in Ts65Dn than in euploids (9, 17, and 7 genes in cerebellum, cortex, and midbrain, respectively); genes whose expression levels partially overlap between the two groups (10, 9, and 14 genes); and genes with intermingled expression, which cannot be used to differentiate trisomics from euploids (12, 5 and 9 genes). Of the genes in the first category, *App*, *Cbr1*, and *Mrps6 *exhibited tight regulation in the three tissues and are therefore attractive candidates for further research.

**Conclusion:**

This is the first analysis addressing inter-individual gene expression levels as a function of trisomy. We propose a strategy allowing discrimination between candidates for the constant features of DS and those genes that may contribute to the partially penetrant signs of DS.

## Abstract 2

RNA from eight Ts65Dn mice (a model of Down syndrome) and eight euploid mice were analysed by real-time PCR to examine inter-individual gene expression levels as a function of trisomy.

## Background

Down's syndrome (DS) is caused by the presence of an extra copy of chromosome 21 (Hsa21) and is the leading genetic cause of mental retardation in the human population. More than 80 clinical features can occur in DS [1-3] that affect virtually all organs of the body. The majority of these features are not present simultaneously in all individuals with DS, and their severity varies considerably from one individual to another.

Analyses of partial trisomies in DS were instrumental in establishing genotype-phenotype correlations [4-6]; however, the notion of a DS critical region (DSCR) has been challenged [7], and this approach failed to identify the genes and associated pathways that contribute to the pathogenesis of DS. Because of inherent problems that limit the use of human samples, a number of molecular and behavioral studies have made use of mouse genetic models of trisomy 21 that reflect some critical phenotypic aspects of DS. The widely studied Ts65Dn model [8,9] parallels several brain-related defects, including quantitative cellular changes in regions of the hippocampus [10,11], reduction in asymmetric synapses in the temporal cortex [12], reduced volume and neuronal density in the cerebellum [13], age-related degeneration of basal forebrain cholinergic neurons [14], and cognitive impairments, especially in tasks mediated by the hippocampus [9,15-17].

It is reasonable to postulate that changes in expression levels of the genes encoded on Hsa21 are primarily responsible for triggering the pathogenesis observed in trisomy. By analyzing RNAs pooled from several Ts65Dn mice in order to minimize inter-individual variation, we and others demonstrated an overall elevation in expression of approximately 1.5-fold for nearly all transcripts of trisomic genes across multiple tissues [18-20]. Bearing in mind that different methodologies were used in these studies (cDNA arrays versus real-time polymerase chain reaction [PCR] with TaqMan or Sybergreen) and that mice at different developmental stages were analyzed, this 1.5-fold elevation in expression is well established as a consistent level in pooled RNAs. A similar magnitude of primary transcript effects was seen in human DS brain and heart for averaged sample values [21]. This level of over-expression is expected under the simplest model of gene regulation, in which transcript level is directly proportional to the gene copy number. More complex patterns could be expected in the case of a trisomic gene that is regulated by a feedback mechanism that involves a downstream product of that gene, or when it is involved in regulatory circuits with the products of other trisomic genes.

Given that changes in gene dosage affect the expression levels of virtually all genes present in three copies, it is reasonable to assume that some of those genes will be neutral for organism fitness, whereas others will exert pathological effects when expression reaches a critical threshold above basal level. However, it is not straightforward to predict which genes will be deleterious when they are over-expressed modestly, even with knowledge of the genes' functions. Among the most pressing questions in DS research are as follows: which genes contribute to specific, constant DS phenotypes (and which do not), and what are the genetic factors that contribute to the phenotypic variability between individuals? Identifying those phenotypes that are associated with differences in gene expression is essential to elucidating the molecular basis of complex traits and disease susceptibility. A recent study [22] showed that as many as 25% of all genes exhibit different expression levels between ethnic groups. Quantitative analyses of gene expression, such as microarrays and quantitative PCR (qPCR), are frequently performed using pooling schemes whose design masks the natural variation of gene expression (sometimes referred as expression phenotype) among individuals. It was previously hypothesized that trisomic genes that exhibit wide variation in expression among individuals with DS would have less impact on the penetrance of the phenotype, which is sin contrast to genes with a moderate variation of expression [23]. Although a few studies conducted in human or mouse have used RNA from individuals rather than from pools [18,20,24,25], this issue of inter-individual variation in gene expression has never directly been addressed from the perspective of candidate genes for DS.

Here, we examine inter-individual differences in expression for 50 mouse orthologs of Hsa21 genes (referred to herein as 'mmu21' genes) in three brain regions of Ts65Dn mice and control littermates (euploids) by means of real-time PCR. This analysis allowed prioritization of the candidate genes for trisomic phenotypes.

## Results

### Expression of mmu21 genes in brain

Transcript levels of mmu21 genes were measured by qPCR in the cerebellum, midbrain, and cortex of eight Ts65Dn and eight control adult mice. Fifty mmu21 genes were tested, of which 33 are triplicated and 17 are disomic in Ts65Dn. Experiments were done in triplicate using two non-mmu21 reference genes that were previously tested for stability in each tissue. For each gene, we calculated a normalized expression value relative to the two reference genes (Additional data file 1).

A large majority of the mmu21 transcripts was found to be active in brain. Forty-two genes (31 trisomic and 11 disomic) were expressed in all three of the brain regions tested. Whereas some of those genes were expressed at high levels in all brain regions (for example, *App*, *Son*, *S100b*, *Itsn*, and *Dyrk1a*), others were differentially expressed (for instance, higher expression of *Sh3bgr*, *Col18a1*, *Tiam1*, *Pde9a*, and *S100b *in cerebellum, and lower expression of *Ncam2 *in cerebellum; data not shown). Eight genes (*Prss7*, *Cryaa*, *Kcne1*, *Fam3b*, *Tff3*, *Tff2*, *Tmprss3*, and *C21orf56*) were excluded from further analysis because they had very low or undetectable levels of expression (for example, Ct > 34 cycles).

### Gene dosage effects in Ts65Dn

**Figure 1:**
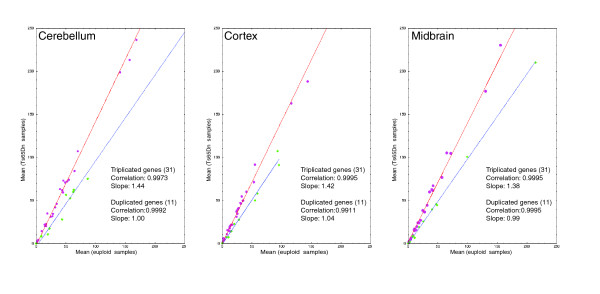
Linear regression plots comparing trisomic and control animals. For each plot corresponding to a given tissue, the linear regression for the triplicated genes is in red, and that for the duplicated genes is in blue. Each gene was plotted using the average of its normalized expressions obtained from the individuals of a group (Ts65Dn on the y-axis and euploid on the x-axis).

**Figure 2:**
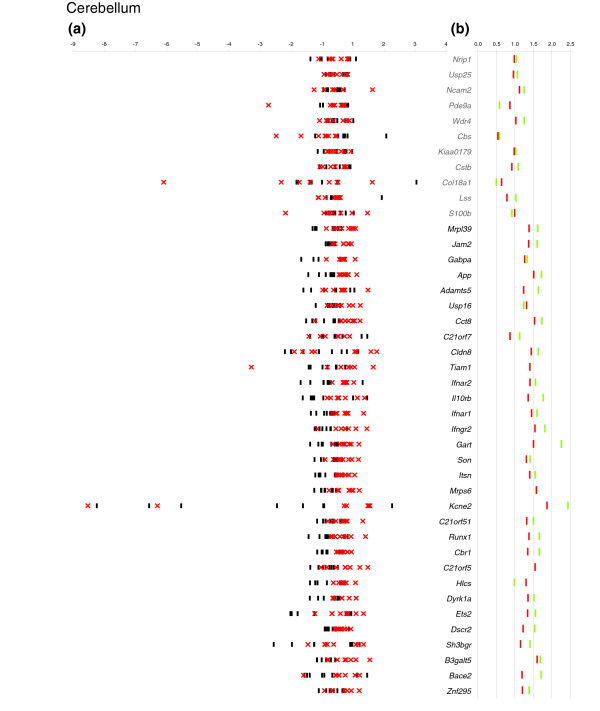
Relative expression and mean Ts65Dn/euploid ratio plots in cerebellum. **(a) **For each of eight Ts65Dn mice (red crosses) and eight euploid mice (black dashes), the log2 ratio of the individual normalized expression over the mean expression across all individuals is plotted on the x-axis. When values for different individuals of a given population are very close, they cannot be distinguished on the graph. On the y-axis each expressed gene is represented in chromosomal order. **(b) **We plotted the mean Ts65Dn/euploid ratios obtained by electronic pooling (red dashes) and the mean Ts65Dn/euploid ratio obtained from a biologic pool (green dashes). The fold changes are given on the x-axis and the gene names on the y-axis. When values for different individuals of a given population are the same, they cannot be distinguished on the graph. Names of genes that are triplicated in Ts65Dn are in bold and disomic genes in grey.

**Figure 3:**
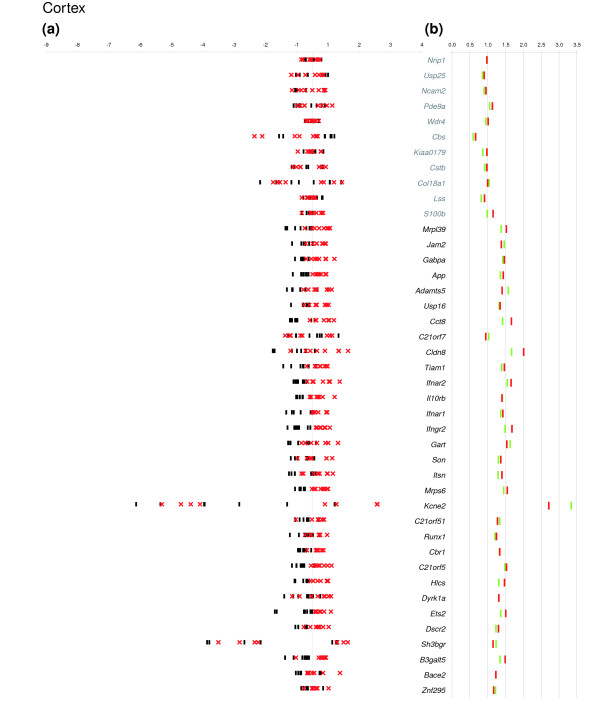
Relative expression and mean Ts65Dn/euploid ratio plots in cortex. **(a) **For each of eight Ts65Dn mice (red crosses) and eight euploid mice (black dashes), the log2 ratio of the individual normalized expression over the mean expression across all individuals is plotted on the x-axis. When values for different individuals of a given population are very close, they cannot be distinguished on the graph. On the y-axis each expressed gene is represented in chromosomal order. **(b) **We plotted the mean Ts65Dn/euploid ratios obtained by electronic pooling (red dashes) and the mean Ts65Dn/euploid ratio obtained from a biologic pool (green dashes). The fold changes are given on the x-axis and the gene names on the y-axis. When values for different individuals of a given population are the same, they cannot be distinguished on the graph. Names of genes that are triplicated in Ts65Dn are in bold and disomic genes in grey.

**Figure 4:**
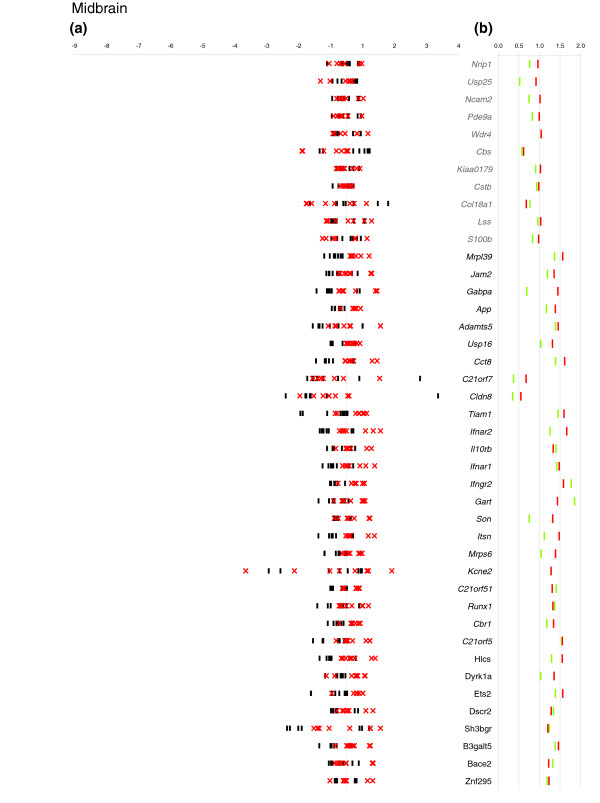
Relative expression and mean Ts65Dn/euploid ratio plots in midbrain. **(a) **For each of eight Ts65Dn mice (red crosses) and eight euploid mice (black dashes), the log2 ratio of the individual normalized expression over the mean expression across all individuals is plotted on the x-axis. When values for different individuals of a given population are very close, they cannot be distinguished on the graph. On the y-axis each expressed gene is represented in chromosomal order. **(b) **We plotted the mean Ts65Dn/euploid ratios obtained by electronic pooling (red dashes) and the mean Ts65Dn/euploid ratio obtained from a biologic pool (green dashes). The fold changes are given on the x-axis and the gene names on the y-axis. When values for different individuals of a given population are the same, they cannot be distinguished on the graph. Names of genes that are triplicated in Ts65Dn are in bold and disomic genes in grey.

For each mmu21 gene, we estimated the gene dosage effect in a given tissue by comparing the ratio of the arithmetic mean of the normalized expression values obtained for the eight Ts65Dn mice with that of the mean of the eight euploids. This value is referred to here as 'electronic' pool (e-pool). We confirmed a trend of 1.5-fold over-expression for the trisomic genes (Figure 1), as reported previously for pooled RNAs from Ts65Dn and controls using arrays or real-time PCR [19]. Assuming that the ratio for diploid genes in trisomic mice versus euploid should be close to 1, the level of over-expression was corrected by the value of slope obtained for the duplicated genes. The global over-expression level of triplicated genes in Ts65Dn was 1.44-fold in cerebellum, 1.37-fold in cortex, and 1.39-fold in midbrain (Figure 1). We also performed a direct measurement of expression levels for RNAs pooled from the eight Ts65Dn and euploid mice, respectively (biologic pools referred as b-pools). We observed a nearly perfect correlation of the b-pools with the e-pools, suggesting that experimental measurement errors are minimal (Figures 2b, 3b, and 4b and Additional data file 2).

Data presented here compare well with previously published findings in Ts65Dn, in which investigations were performed in an independent set of mice and with different chemistry (TaqMan versus SYBR green or cDNA arrays) [19]. Indeed, we observed a global correlation of 80% between the two studies comparing the Ts65Dn/euploid ratios for pooled RNAs using binned value ranges (see Materials and Methods). A direct comparison could not be made with the study of Lyle and coworkers [18] because they measured expression profiles in whole brain of RNAs from Ts65Dn mice at a different age. However, they also reported an overall over-expression close to 1.5-fold for the trisomic genes in Ts65Dn.

We used pooling schemes with eight individual Ts65Dn and control mice as a prerequisite to assess the robustness of our measurements comparing e-pools and b-pools. We validated the 1.5-fold over-expression of trisomic genes in Ts65Dn. However, this value represents a global trend that does not exhibit potential variations in gene expression between individuals. In the next step, we investigated the variation of expression levels for the mmu21 genes in the eight trisomic and control mice.

### Variation of gene expression in the brains of Ts65Dn and control mice

Analysis of individual samples allows recovery of important information that cannot be determined from pooled samples. We estimated the variation of gene expression between individual mice by using the coefficient of variation (CV). We then assessed whether the two populations (Ts65Dn and euploid) differ significantly in terms of this variance by using the F-test, and we applied the Wilcoxon test to judge whether the differences in expression levels between Ts65Dn and euploid animals were significant. Data are summarized in Additional data file 3.

It was first important to evaluate the potential influence of technical variation as compared with that of biological variation. We calculated technical variance based on the experimental replicates and biological variance measured between individuals in each group (Ts65Dn or euploid; see Materials and methods [below] and Additional data file 3), similar to analysis of variance estimations. We considered the percentage of technical variance over the total variance (technical + biologic variance) as a quality measure. The inter-individual variation in gene expression observed here can be attributed mostly to biologic differences because for most genes the biological variance contributes to more than 90% of the total variance (Additional data file 3). We could reliably detect expression differences as small as 1.3-fold for nearly all of the assays, because the average standard error of the mean for the relative expression measurements was 0.14 in the 95% confidence intervals.

We used the CV (see Materials and methods [below] and Additional data file 3) as an indicator of the variation in gene expression among individual mice in each group (Ts65Dn or euploid). The CV, in contrast to the variance, is independent of the gene expression level and thus enables inter-gene comparison. To group the CVs, we set arbitrary cut-offs defined as a very low (CV < 0.1), low (CV < 0.2), moderate (CV between 0.2 and 0.5), and high (CV > 0.5) variation in gene expression. In control mice six genes exhibited a CV < 0.1, suggesting that they are tightly regulated (*Jam2 *in cerebellum; *App*, in midbrain; and *App*, *Wdr4*, *S100b*, *Gabpa*, and *Mrps6 *in cortex). *App *was previously reported to exhibit highly variable expression in lymphoblastoid cell lines [26], but it appears here to be tightly regulated, with a CV of 0.18 in cerebellum, 0.1 in cortex, and 0.09 in midbrain. Few genes (three, five, and nine genes in cortex, midbrain, and cerebellum, respectively) exhibit highly variable expression levels (CV > 0.5). In Ts65Dn mice, 24 out of the 31 triplicated genes have CVs below 0.2, suggesting that although their expression is elevated, it remains relatively tightly regulated. None of the triplicated genes had CVs below 0.1. Only four triplicated genes (*Kcne2*, *C21orf7*, *Cldn8*, and *Sh3bgr*) exhibit a systematic high fluctuation in expression levels (CV > 0.5) across individuals and tissues. Among the disomic genes, two were tightly regulated (CV < 0.1) in cortex (*Wdr4 *and *Lss*) and one in the midbrain (*Cstb*). One disomic gene (*Col18a1*) exhibited high variation in expression levels (CV > 0.5) among all brain regions and in both groups (Ts65Dn and euploid).

For all of the trisomic genes, we plotted CVs for the Ts65Dn population against the CVs for the euploid population (Additional data file 4). For most of the tested genes there is no significant difference in the variation of expression levels between trisomic and euploid mice, because 85 out of 93 data points do not differ by more than 20% in CVs between the two groups. On average, 90% of the trisomic genes exhibit low or moderate variation in expression among groups. A few outliers, such as *Kcne2 *and *C21orf7*, exhibit dramatic variations in expression among mice. *Kcne2*, for example, was found to be highly variable in all three brain tissues and in both groups (Ts65Dn and euploid).

The variance in expression levels increases as gene expression increases. This obscures the fact that, in proportion, the level of variation is not greater. To determine statistically whether the presence of an extra copy of a gene influences its variation in expression level, we used the CV calculated with the F-test to circumvent this artefact. We observed that, overall, the amplitude of variation of gene expression did not differ significantly (*P *> 0.05) between euploid and Ts65Dn mice (Additional data file 3). The basal level of expression of a given gene follows a Gaussian distribution of similar width in trisomic and in euploid mice. This result confirms earlier findings in fetal cortex of human trisomy 21 [24], at least as far as mmu21 genes are concerned. Three trisomic genes appeared to exhibit variation that was significantly greater (*P *< 0.05) in Ts65Dn than in euploid mice (*Gabpa *and *Ifnar2 *in the cortex, *B3galt5 *in the cerebellum). Contrary to our expectations, however, the CV was significantly smaller (*P *< 0.05) in trisomic mice for five trisomic genes (*Ifnar2 *and *Kcne2 *in cerebellum, *Ets2 *in cortex, and *Usp16 *and *Cldn8 *in midbrain). Moreover, we see few disomic genes with significant differences in CV (*P *< 0.05) between the two groups in the cerebellum; for *S100b *and *Ncam2 *the CV increases in Ts65Dn mice, whereas it decreases for *Lss*.

The expression levels of mmu21 genes in individual mice are shown in Figures 2a, 3a and 4a, where we plotted for each tissue the log ratios of the individual normalized gene expression values over the mean expression across all of the eight mice. For the purposes of direct comparison, the corresponding Ts65Dn/euploid gene expression ratios obtained from e-pools and b-pools are indicated on the same figure for each tissue (Figures 2b, 3b, and 4b). The figures enable identification of the genes with expression that varies substantially from those that are tightly regulated, and they demonstrate whether the corresponding mean ratio reflects the variability in gene expression in each tissue.

Finally, we evaluated the significance of the differences in expression levels between Ts65Dn and euploid mice by applying the nonparametric Wilcoxon test, which is robust against outlier values (Additional data file 3). For instance, in the midbrain the Ts65Dn/euploid ratio values of two trisomic genes are downregulated; these genes are *C21orf7 *(e-pool: ×0.68; b-pool: ×0.37) and *Cldn8 *(e-pool: ×0.55; b-pool: ×0.35; Figure 4). However, the distribution of the expression levels among single mice is broadly dispersed. In these particular cases, the high CV values in each group (Additional data file 3) and the nonsignificant *P *values of the Wilcoxon test (*P *> 0.05) confirmed that pooled Ts65Dn/euploid ratios did not reflect a genuine trend toward down-regulation at the level of individual mice. However, it should be noted that this gene, like *Cldn8*, is expressed at very low levels in brain. Similar effects are observed for these genes in cerebellum and cortex.

Three trisomic genes that were previously reported not to conform to the 1.5× rule, namely *Bace2 *in the cortex (×2.15), *Kcne2 *(×3.39), and *Sh3bgr *(×0.55) in midbrain [19], fall into the 1.5-fold range when analyzed as either b-pool or e-pool (Figures 2b, 3b, and 4b). Interestingly, these genes are among those that exhibit high variability in expression levels among groups in at least one tissue (Figures 2a, 3a, and 4a). The most extreme case of inter-individual variation in expression was observed for *Kcne2*, for which the CV values where always above 0.5 in all groups and all tissues. Both b-pool and e-pool ratios for *Kcne2 *are in the 1.5-fold range in the midbrain, whereas the apparent higher order upregulation in the cortex (e-pool: ×2.71; b-pool: ×3.34), does not reflect the individual expression levels spanning the largest range (Figures 2, 3, and 4). We observed that genes for which Ts65Dn/euploid ratios are skewed in pooled RNAs are most often those with high expression variation differences between mice. We conclude from this that the pool value is at best indicative of high variation in gene expression level across mice, but that it does not necessarily reflect a genuine upregulation or downregulation.

Two of the mmu21 disomic genes exhibited altered expression in our analysis. As in our previous study [19], *Cbs *RNA levels were reduced in cerebellum (e-pool: ×0.54; b-pool: ×0.58), midbrain (e-pool: ×0.62; b-pool: ×0.58), and cortex (e-pool: ×0.66; b-pool: ×0.59). For *Cbs*, a consistent and significant trend toward downregulation (Figures 2, 3, and 4) can be observed in all three brain tissues in individual mice. *Cbs *is involved in the transsulfuration pathway and converts homocysteine to cystathionine. *Cbs *deficiency can cause homocysteinuria, which affects the central nervous system among many other organs. Our data strongly suggest that *Cbs *is downregulated in response to trisomy in Ts65Dn, although we cannot determine whether it is directly or indirectly regulated by other mmu21 genes.

Pool data indicated that *Col18a1 *was reduced in cerebellum (e-pool: ×0.64; b-pool: ×0.50) and in midbrain (e-pool: ×0.68; b-pool: ×0.77). However, individual gene expression levels for *Col18A1 *were broadly dispersed in these tissues, and the two groups (Ts65Dn and euploid) are not clearly distinguishable (*P *> 0.05).

### Stratification of trisomic genes based on brain gene expression profiles

Three main categories of trisomic genes can be distinguished from the normalized expression levels: genes whose expression levels in the eight Ts65Dn mice are clearly above those in their euploid littermates (for instance, *App *in cortex or *Mrlp39 *in midbrain), enabling a clear distinction between the two populations; genes whose expression levels partially overlap between the two populations of mice (for example, *Runx1 or C21orf5 *in cerebellum); and genes with intermingled expression between the two populations (for instance, *Bace2 *or *Kcne2 *in cerebellum), which do not distinguish Ts65Dn from euploid animals. As expected, all of the 11 disomic genes tested here were found to be in the latter category except *Cbs *(see above).

**Figure 5:**
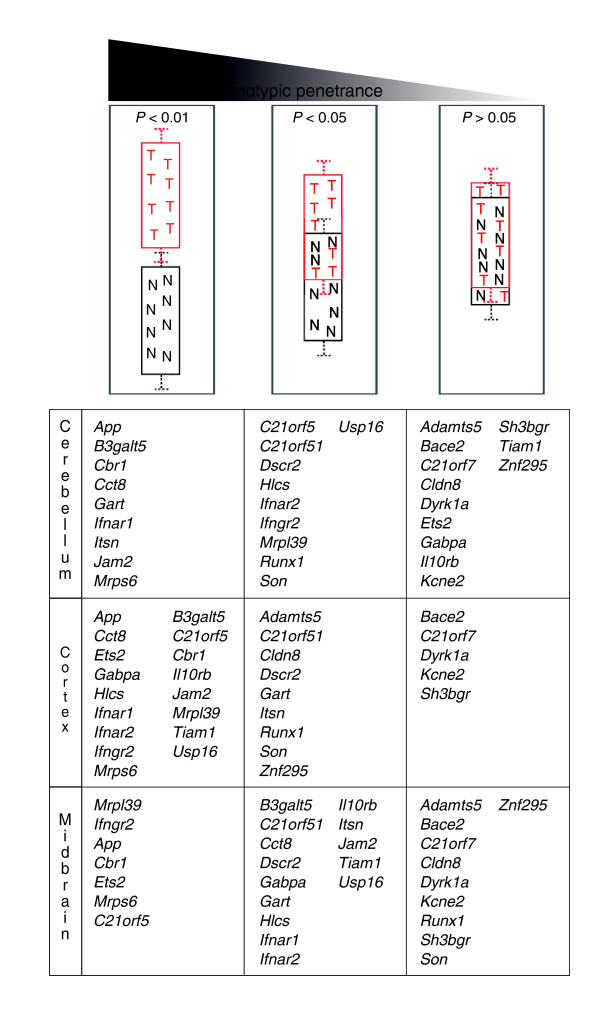
Gene categorization by phenotype penetrance. Genes are grouped in three categories, according to *P *value (Wilcoxon test) and the tissues in which they were tested. The first category (left) shows genes with *P *< 0.01, meaning that the expression levels in Ts65Dn individual mice are consistently different from euploids. The second category shows genes with 0.01 <*P *< 0.05, for which the expression levels of Ts65Dn samples partially overlap with euploids. The last category (*P *> 0.05) groups genes for which the expression levels between Ts65Dn and euploid mice cannot be distinguished. Genes in the first category might be responsible for the fully penetrant signs in trisomy, genes in the second could contribute to the variable signs, whereas the third category contains genes that may make little or no contribution.

Based on the distribution of expression levels in the brain, the three categories of mmu21 genes at dosage imbalance are presented in Figure 5. The first category contains genes whose expression in an individual trisomic mouse was always significantly higher than in any euploid animal tested (*P *< 0.01). It should be noted that in this first category we observed that, for some genes, one individual out of the trisomic or control group exhibited a slight overlap in its transcript level with that of the second genetic group, although it was still associated with consistent and significantly elevated expression levels in one group of mice as compared with the other (*P *< 0.01; for example, *Jam2 *in cerebellum; Figure 2). Across the three brain tissues, 20 genes exhibit expression levels significantly higher in trisomic than in euploid mice. We speculate that genes in this first category may have a greater penetrance in the cerebellar phenotypes observed in mouse models of DS [27-29] and may also be important candidates in structural and functional deficits in the DS brain. Notably, three genes (*App*, *Cbr1*, and *Mrps6*) belong to this highly differentiated category for all three brain regions. Furthermore, many genes in this category (for instance, *Jam2*, *App*, *Cbr1*, *Cct8*, *Itsn*, *Mrps6*, and *C21orf5*) are conserved at least in *Caenorhabditis elegans *and *Drosophila melanogaster*, and are tightly regulated with a CV below 20% in trisomic or euploid, or both, indicating that these genes are important for development of the organism. Two genes that are good candidates for neurodegenerative pathologies associated with DS are *App*, which is mutated in some forms of Alzheimer's disease [30] and of which the upregulation contributes substantially to degeneration of cholinergic neurons [31] and *Itsn *(Intersectin), which is involved in clathrin-mediated endocytosis [32]. *Jam2 *is associated with cell adhesion processes and *Cct8 *with protein folding and degradation. *C21orf5 *is a new member of the Dopey family and exhibits restricted regional brain expression [33].

The second category is populated by trisomic genes for which expression levels are partially or totally intermingled between the two populations of mice (*P *values between 0.01 and 0.05). Overall, 22 genes are included in this category. However, of these only two genes are found in all three brain tissues (*C21orf51 *and *Dscr2*). In general, genes in this category exhibit moderate variation in expression based on the CV. We hypothesize that genes whose expression is more variable are more likely to contribute to those pathogenic outcomes in DS that exhibit variable expressivity.

The third class includes genes for which the expression levels are intermingled between the two populations of mice (*P *> 0.05). Overall, 15 genes are included in this last category, and among these five were found in the three tissues (*Bace2*, *C21orf7*, *Dyrk1a*, *Kcne2*, and *Sh3bgr*). They all have moderate to high expression variation based on the CV. Thus, those genes may be less likely to contribute to constant trisomy-related phenotypes. Genes whose expression levels vary widely among individuals are more likely to reach a critical threshold of over-expression in a subset of individuals, and thus they might be more likely to be involved in phenotypes that occur in some but not all individuals with trisomy 21. We also observed notable behaviour differences between tissues. Four genes (*Ets2*, *Gabpa*, *Il10rb*, and *Tiam1*) were found in the first category for cortex, but were allocated to the third category for cerebellum. Notably, in midbrain *Ets2 *was also found in the first category whereas *Gabpa*, *Il10rb*, and *Tiam1 *were found in the second category. Either these genes are tightly controlled in a tissue specific manner or more samples must be analysed to assign them to one or the other group with certainty.

## Discussion

It is widely accepted that gene products at dosage imbalance are the primary contributors to the trisomy phenotypes, acting either directly or indirectly via disturbance of complex regulatory networks. Characterizing these primary changes at the transcriptome level is a first essential step toward the identification of affected biochemical pathways associated with trisomy 21.

We measured expression levels of 33 trisomic and 17 disomic mmu21 genes in eight adult Ts65Dn and in eight euploid mice to identify inter-individual variations in expression and whether they were affected by trisomy. The simplest model of trisomic gene actions predicts that expression level is proportional to gene copy number. This '1.5× rule' was substantiated by examining pooled RNAs in multiple tissues from several individuals in independent analyses [18,19]. In the present study we also assessed Ts65Dn/euploid gene expression ratios in pools and corroborated previous findings using pooled samples; specifically, most trisomic genes exhibit an increased transcript level that is about 50% higher than in euploid across tissues. Several specific instances of trisomic genes that do not follow this rule were previously reported [18,19]. Here we investigated the possibility that genes that did not adhere to the expected 1.5-fold trend could arise from the natural inter-individual gene expression variation by evaluating the amplitude of gene expression at every tested locus in RNA samples from individual mice.

Evaluation of individuals revealed variation of gene expression to fall in the range of 20% to 50% for most of the mmu21 genes, whereas only a few genes exhibit either tight regulation (<20% variation in expression among individuals) or dramatically different expression levels across individuals. Consequently, Ts65Dn/euploid ratios must be interpreted with caution. For instance, three genes that were previously shown to escape the 1.5× rule, namely *Bace2 *in cortex and *Kcne2 *and *Sh3bgr *in midbrain, exhibit wide inter-individual variation, which could account for these skewed ratios. Assessment of gene expression levels in individuals also provided further evidence for dysregulation of the disomic gene *Cbs *in the three brain regions of Ts65Dn, regardless of the inter-individual variation observed for *Cbs*.

Analyses of pooled RNAs minimize inter-individual variations and have been useful in providing an averaged measure of over-expression level in trisomic tissues and to identify possible outliers. Nonetheless, pooling schemes are also associated with intrinsic errors (for instance, slight differences in the RNA amount added to the pool), which may contribute to additional variance. Even though methods have been proposed for accurate analysis of data from large-scale DNA pools [34], Ts65Dn/euploid ratios must be interpreted in conjunction with the distribution of the gene expression values in trisomic and euploid individuals. We showed that limitations to the technique (real-time PCR) were unlikely to be a significant factor, because the techniques are sufficiently sensitive to detect differences in expression values that are substantially smaller than those observed.

As in most expression profiling studies, our data represent a snapshot of the expression level in one individual at the time of death. We cannot exclude the possibility that the expression of some genes may be sensitive to the local environment (for example nutrition, temperature, stress, and light). Inherent individual variations in the 'personal statistics' of the mouse (weight, size, metabolite levels, for example), all of which affect the number and proportions of cell types in tissues and organs, may lead to changes in the RNA population as well. Some of the variation in expression that we observe may also reflect variation over time or cycling of gene expression levels. However, it is unlikely that cells are synchronized throughout a complex tissue, and such effects are expected to be averaged out for most genes. Ts65Dn is maintained as an advanced intercross between the inbred B6 and C3H strains and thus have some variability in genetic background. We expect that the genetic contribution to differences in expression phenotypes is not large between strains of mice that are relatively closely related (C3H and B6), and euploid/trisomic variation in genetic background is further reduced by using littermate pairs. Nonetheless, allelic variation between individual Ts65Dn mice still represents a factor that must be taken into account. At the same time, variations seen consistently on the more robust genetic background are likely to be representative of 'real' populations. Any or all of these factors may contribute to the observed variation.

We, as have others previously [23], posit that this variation in gene expression, which was masked in pools, may provide insights into those genes that are involved in constant or variable features of DS, especially when considered in light of a threshold effect for gene dosage. Of course, the operative mechanism will involve the actual quantity of a gene product in a cell. This may become pathogenic once it passes a specific threshold (or drops below a minimum concentration that is necessary for its function). Although evolution has allowed rather loose control of the expression of some genes, others are under tight constraint. For example, it has been shown in primates that expression level control is crucial during evolution, and that genes with higher inter-species and intra-species variation will give rise to different functions and effects more often than those that are very tightly controlled [35]. What is not clear, however, is the level of over-expression relative to the normal state that can be tolerated without ill effects for a specific gene product, or how sharp the onset of possible deleterious effects of over-expression could be.

Our results demonstrate the importance of considering gene expression in individuals, and this approach is even more important in human samples, which exhibit greater genetic background heterogeneity than do Ts65Dn mice. Normal variation in gene expression plays a role in susceptibility to complex diseases and likewise plays a potentially relevant role in the phenotypic differences seen between individuals with DS. Although DS presents highly variable clinical features, some phenotypes are common to all, irrespective of genetic background. We expect that these common features derive from dysregulated gene expression that exhibits the same pattern in all individuals. Here we identified three classes of genes with different expression levels relative to euploid. The first class is populated by genes whose expression levels are significantly higher in trisomic than in euploid individuals. The second class is represented by genes with partially overlapping expression levels between the two populations, whereas genes with high degrees of intermingled expression levels form the third class. We postulate that genes in the first class represent good candidates for the constant phenotypes of DS.

In this first, class *Cct8 *and *Ifnar1 *appeared to be tightly regulated in cerebellum and cortex. In a study investigating gene expression variation in 40 human lymphoblastoid cells lines [26], *CCT8 *and *IFNAR1 *also appeared to be tightly controlled. This observation shows that, despite the fact that different tissues were investigated, and given that lymphoblastoid cell lines may not always reflect the situation in primary tissues, the use of mouse tissue can be predictive of gene behavior in human. However, we should keep in mind (as discussed above) that even within a given organism, a specific gene may appear tightly regulated in one tissue but not in another. Also, we cannot exclude the possibility that this variation pattern could change during development, but no data are currently available to address this latter issue.

Three genes from the first category are common to cerebellum, cortex, and midbrain (*App*, *Cbr1*, and *Mrps6*), identifying these as strong candidate genes for Ts65Dn neuroanatomic defects. Along this line, Salehi and colleagues [31] recently reported evidence for a pathogenic mechanism for DS in which increased expression of *App *(which encodes the amyloid precursor protein) causes abnormal transport of nerve growth factor, resulting in cholinergic neurodegeneration in a mouse model of DS. In contrast, the genes whose trisomic expression levels overlap completely with euploid appear less likely to be key players in the invariant features of trisomy. Among those, we found that *Kcne2 *and *Sh3bgr *exhibited a dramatic variation in expression level regardless of ploidy. Expression levels for a number of genes fell between these two extremes, as represented by the second category. This may indicate the limit of precision for this method, but it could also represent a pool of candidates for partially penetrant phenotypes. If the disomic level of a given gene is close to a critical threshold, then elevated gene expression might be deleterious only to those trisomic individuals with the highest expression, contributing to variability in the occurrence of DS features.

This approach provides a logical strategy for prioritizing candidate genes that are likely to contribute to the brain phenotypes observed in Ts65Dn. The present analysis should be consolidated further by an exhaustive expression analysis in a large number of individuals at several stages of development. It may be that the deleterious effect of overproduction of gene products occurs mostly at a specific place and time during development, when the level of the gene product is particularly high. It also appears that variability in the levels of the expression of a specific gene is a true characteristic of some genes, which must be considered in a description of how elevated expression of a particular gene contributes to pathogenesis in DS. The level of mRNA does not systematically reflect the downstream protein amount but it is a good indicator. Moreover, the lack of methods that are sensitive enough to measure subtle differences in protein levels at a large scale justifies the strategy used here.

Starting from the postulate that most of the trisomic genes are over-expressed by a factor of 1.5, speculations on candidate genes were initially based on the molecular function of the genes. Favorite candidates include, for instance, tightly regulated gene products that exert trans effects, such as the following: transcription factor complexes that establish concentration gradients during development; molecules that are involved in epigenetic mechanisms that modulate the accessibility of DNA to the transcriptional machinery; receptor-ligand-signal transduction systems; and proteins that modulate the activity of other proteins. However, many genes play a pivotal role in various cellular processes, and it is difficult to identify dosage-sensitive genes *a priori*. Dissection of the molecular basis for aneuploid phenotypes will require a massive body of information that is still largely incomplete, including detailed gene expression patterns within developing organisms [33] and knowledge of genome-wide genetic networks, as well as allelic contributions to variability in the level, place and time of expression, and the variation in basal gene expression levels in the population. An understanding of the pathogenesis that produces features of DS will require integration of this type of gene expression data with a quantitative description of variable phenotypic outcomes in DS. Mapping the regulators of Hsa21 genes in man and in mouse is essential to elucidating the genetic basis of the variation of gene expression and its contribution to pathogenesis in DS.

As shown here, stratification of populations by expression profiling provides an essential dimension in the molecular analysis of aneuploidy syndromes. Identifying the pathways that are perturbed by trisomy will require thorough studies of expression phenotypes at the level of a global transcriptome, and integration of other large-scale experiments designed to decipher gene regulatory networks.

## Conclusion

The issue of natural gene expression variation in trisomy 21 has not previously been addressed directly, and to the best of our knowledge this is the first report dealing with this important issue, an appreciation of which is essential to our understanding of DS and aneuploidies in general. We describe a strategy based on variation in gene expression to stratify the chromosome 21 genes (or their orthologs), which are candidates for the trisomy phenotypes. This is a novel dimension in the search of culprit genes for DS that enables one to propose a short list of putative candidates among the genes at dosage imbalance.

## Materials and methods

### RNA extraction and reverse transcription

Total RNA was extracted from frozen tissues of Ts65Dn males and their euploid male littermates using Trizol reagent (Invitrogen, San Diego, CA, USA), following the manufacturer's instructions. Animals were between 13 and 16 weeks old. RNA was treated with RNase-free Dnase I, quantified by UV spectrophotometry, and its integrity was verified by gel electrophoresis. To create the sample pools, equal amounts of RNAs from four euploid or four trisomic animals for each brain tissue were pooled. RNA was transcribed into cDNA using random hexamers and SuperscriptII reverse transcriptase (Invitrogen). In total, 8 μg total RNA for each sample was converted into cDNA in 8 × 1 μg reactions, pooled, and diluted to 12.5 ng/μl equivalent total RNA.

### Quantification strategy

For quantitative gene expression studies, we used pre-designed, gene-specific TaqMan^® ^probe and primer sets provided by Applied Biosystems (Foster City, CA, USA) (references for each assay are given in Additional data file 1). All assays met the amplification efficiency criteria of 100% ± 10% (ApplicationNote 127AP05-02 [36]) and were comparable to each other. For normalization purposes, 18 non-mmu21 control genes were tested on sample cDNA. We identified the most stable genes across samples using the geNorm method [37]. Thus, two genes (*Hprt *and *Hmbs*; Additional data file 1) were selected and data were normalized to their geometric mean. All assays were performed in triplicate. To minimize intra-assay variation, the sample cDNA was premixed with the PCR mastermix and distributed equally into each reaction. For a given target gene all tissue samples were run on the same reaction plate. This increases the accuracy of inter-individual comparison, because the mRNA of interest is amplified under the same PCR conditions in all tissue samples. To validate the reproducibility of our system, one experiment including two cDNA samples was duplicated. The correlation between the two independent experiments was in excess of 99% (data not shown).

### Real-time quantitative polymerase chain reaction

All reactions were set up in 10 μl volumes and used the TaqMan Universal PCR Master Mix (Applied Biosystems, Foster City, CA, USA). Assays were processed with the ABI Prism 7900HT Sequence detection System (Applied Biosystems, Foster City, CA, USA) under the following conditions: 50°C for 2 min, 95°C for 10 min, and 40 cycles of 95°C for 15 s/60°C for 1 min. Amplification plot and predicted threshold cycle (Ct) values were obtained with the sequence Detection Software (SDS 2.1; PE Applied Biosystems). Further calculations and graphical representations were done using Excel 2000. We verified that no correlation could be found between threshold cycles (Ct) and expression ratios (Ts65Dn/euploid), indicating that there was no systematic biases within our real-time PCR results. Nonetheless, It should be noted that when the Ct value increases above about 32, the standard error also increases, indicating a loss of precision of the replicate measurements.

### Data analysis

A common threshold value was chosen for all genes and the baseline was set manually for individual genes. The relative expression calculation method relies on the principle of the comparative Ct method (User Bulletin #2; Applied Biosystems). Ct values were first normalized (ΔCt) to a geometric mean of the two normalization genes and converted to a relative expression quantity (NE) using the formula NE = 2^-ΔCt^. A given Ts65Dn/euploid ratio was calculated by dividing their respective NE values. For electronic pool (e-pool) calculation, the NE values for all individuals in a given group (Ts65Dn or euploid) were averaged. For the analysis presented here, we considered binned ratios of 0.8 to 1.2 to be neutral (no change in expression), whereas binned values from 1.2 to 2.0 were considered equivalent to 1.5-fold expression change.

In order to compute variation across a sample, we calculated the coefficient of variation:

CV=σNE¯
 MathType@MTEF@5@5@+=feaafiart1ev1aaatCvAUfeBSjuyZL2yd9gzLbvyNv2Caerbhv2BYDwAHbqedmvETj2BSbqee0evGueE0jxyaibaiKI8=vI8tuQ8FMI8Gi=hEeeu0xXdbba9frFj0=OqFfea0dXdd9vqai=hGuQ8kuc9pgc9s8qqaq=dirpe0xb9q8qiLsFr0=vr0=vr0dc8meaabaqaciGacaGaaeqabaqadeqadaaakeaacaqGdbGaaeOvaiabg2da9maalaaabaaccaGae83WdmhabaWaa0aaaeaacaqGobGaaeyraaaaaaaaaa@3954@

Here, NE¯
 MathType@MTEF@5@5@+=feaafiart1ev1aaatCvAUfeBSjuyZL2yd9gzLbvyNv2Caerbhv2BYDwAHbqedmvETj2BSbqee0evGueE0jxyaibaiKI8=vI8tuQ8FMI8Gi=hEeeu0xXdbba9frFj0=OqFfea0dXdd9vqai=hGuQ8kuc9pgc9s8qqaq=dirpe0xb9q8qiLsFr0=vr0=vr0dc8meaabaqaciGacaGaaeqabaqadeqadaaakeaadaqdaaqaaiaab6eacaqGfbaaaaaa@34D7@ is the mean expression and σ is the standard deviation of eight samples in a group.

In order to estimate the technical variance (TechVar) and the biological variance (BioVar) for each gene across the different individuals and the different technical replicate measurements, we used an analysis of variance-like approach:

TechVar=∑i=1I∑j=1J(NEij−NE¯i.)2
 MathType@MTEF@5@5@+=feaafiart1ev1aaatCvAUfeBSjuyZL2yd9gzLbvyNv2Caerbhv2BYDwAHbqedmvETj2BSbqee0evGueE0jxyaibaiKI8=vI8tuQ8FMI8Gi=hEeeu0xXdbba9frFj0=OqFfea0dXdd9vqai=hGuQ8kuc9pgc9s8qqaq=dirpe0xb9q8qiLsFr0=vr0=vr0dc8meaabaqaciGacaGaaeqabaqadeqadaaakeaacaqGubGaaeyzaiaabogacaqGObGaaeOvaiaabggacaqGYbGaeyypa0ZaaabCaeaadaaeWbqaaiaacIcacaqGobGaaeyramaaBaaaleaacaqGPbGaaeOAaaqabaGccqGHsisldaqdaaqaaiaab6eacaqGfbaaamaaBaaaleaacaqGPbaabeaakiaac6caaSqaaiaabQgacqGH9aqpcaaIXaaabaGaaeOsaaqdcqGHris5aOGaaiykamaaCaaaleqabaGaaGOmaaaaaeaacaqGPbGaeyypa0JaaGymaaqaaiaabMeaa0GaeyyeIuoaaaa@5041@

BioVar=J∑i=1I(NEi¯.−NE¯)2
 MathType@MTEF@5@5@+=feaafiart1ev1aaatCvAUfeBSjuyZL2yd9gzLbvyNv2Caerbhv2BYDwAHbqedmvETj2BSbqee0evGueE0jxyaibaiKI8=vI8tuQ8FMI8Gi=hEeeu0xXdbba9frFj0=OqFfea0dXdd9vqai=hGuQ8kuc9pgc9s8qqaq=dirpe0xb9q8qiLsFr0=vr0=vr0dc8meaabaqaciGacaGaaeqabaqadeqadaaakeaacaqGcbGaaeyAaiaab+gacaqGwbGaaeyyaiaabkhacqGH9aqpcaqGkbWaaabCaeaacaGGOaWaa0aaaeaacaqGobGaaeyraiaabMgaaaGaaeOlaiabgkHiTmaanaaabaGaaeOtaiaabweaaaGaaiykamaaCaaaleqabaGaaGOmaaaaaeaacaqGPbGaeyypa0JaaGymaaqaaiaabMeaa0GaeyyeIuoaaaa@4825@

Where I is 8 (the size of the samples group), J is 3 (the technical replicate number), NE_ij _is the j-th replicate measurement in the i-th individual, NE¯i.
 MathType@MTEF@5@5@+=feaafiart1ev1aaatCvAUfeBSjuyZL2yd9gzLbvyNv2Caerbhv2BYDwAHbqedmvETj2BSbqee0evGueE0jxyaibaiKI8=vI8tuQ8FMI8Gi=hEeeu0xXdbba9frFj0=OqFfea0dXdd9vqai=hGuQ8kuc9pgc9s8qqaq=dirpe0xb9q8qiLsFr0=vr0=vr0dc8meaabaqaciGacaGaaeqabaqadeqadaaakeaadaqdaaqaaiaab6eacaqGfbaaaiaabMgacaqGUaaaaa@3674@ = mean NE for the ij-th individual, and NE¯
 MathType@MTEF@5@5@+=feaafiart1ev1aaatCvAUfeBSjuyZL2yd9gzLbvyNv2Caerbhv2BYDwAHbqedmvETj2BSbqee0evGueE0jxyaibaiKI8=vI8tuQ8FMI8Gi=hEeeu0xXdbba9frFj0=OqFfea0dXdd9vqai=hGuQ8kuc9pgc9s8qqaq=dirpe0xb9q8qiLsFr0=vr0=vr0dc8meaabaqaciGacaGaaeqabaqadeqadaaakeaadaqdaaqaaiaab6eacaqGfbaaaaaa@34D7@ is the overall mean NE for all samples and technical replicates.

The total variance (TotVar) is defined by the following equations:

TotVar=∑i=1I∑j=1J(NEij−NE¯i)2
 MathType@MTEF@5@5@+=feaafiart1ev1aaatCvAUfeBSjuyZL2yd9gzLbvyNv2Caerbhv2BYDwAHbqedmvETj2BSbqee0evGueE0jxyaibaiKI8=vI8tuQ8FMI8Gi=hEeeu0xXdbba9frFj0=OqFfea0dXdd9vqai=hGuQ8kuc9pgc9s8qqaq=dirpe0xb9q8qiLsFr0=vr0=vr0dc8meaabaqaciGacaGaaeqabaqadeqadaaakeaacaqGubGaae4BaiaabshacaqGwbGaaeyyaiaabkhacqGH9aqpdaaeWbqaamaaqahabaGaaiikaiaab6eacaqGfbWaaSbaaSqaaiaabMgacaqGQbaabeaakiabgkHiTmaanaaabaGaaeOtaiaabweaaaGaaeyAaiaacMcadaahaaWcbeqaaiaaikdaaaaabaGaaeOAaiabg2da9iaaigdaaeaacaqGkbaaniabggHiLdaaleaacaqGPbGaeyypa0JaaGymaaqaaiaabMeaa0GaeyyeIuoaaaa@4E7F@

TotVar = TechVar + BioVar

For judging variance differences in the trisomic and euploid samples, we applied an F-test for each gene. This test requires the two samples to have a Gaussian distribution with the same mean value. Therefore, we divided each individual observation from a sample by the mean value of that sample group prior to analysis. Small *P *values in the F-test indicate whether there is a significant difference in expression variation.

For each gene we performed statistical tests based on the replicate signals in experiments with trisomic and euploid samples. Three standard tests were used in parallel: Student's *t*-test, the Welch test, and Wilcoxon's rank-sum test. To evaluate differential expression of the genes, *P *values of Wilcoxon's rank-sum test were preferred as a reference because this test does not depend for its validity on a specific distribution (for example, Gaussian). Furthermore, this test is robust against outlier values in the sample. A recursive function was implemented to calculate the precise *P *values [38].

## Additional data files

The following data are available with the online version of this paper. Additional data file 1 provides a table listing the references of the gene expression assay (Applied Biosystems) that were used for quantitative RT-PCR experiments. Additional data file 2 provides a figure of the correlation plot of intensities from electronic pools (y-axis) versus biologic pools (x-axis) for each gene in the three brain tissues. Additional data file 3 is a summary table listing the following information for each gene and each brain tissue analyzed: gene names (triplicated genes in Ts65Dn are in red), mean expressions (ME) of Ts65Dn and euploid mice, standard errors of MEs, CVs of the Ts65Dn and euploid samples, technical and biological variance, mean trisomic:euploid gene expression ratios from electronic and RNA pools, and *P *values from *t*-test, *tu*-test, Wilcoxon test, permutation test, and F-test. Additional data file 4 is a scatter plot of CVs of euploid versus Ts65Dn mice in brain tissues, in which the dotted lines represent the ± 20% CV deviations from the ideal correlation (plain line).

## Supplementary Material

Additional data file 1 Provided is a table listing the references of the gene expression assay (Applied Biosystems) that were used for quantitative RT-PCR experiments. Click here for file

Additional data file 2 Provided is a figure of the correlation plot of intensities from electronic pools (y-axis) versus biologic pools (x-axis) for each gene in the three brain tissues. Click here for file

Additional data file 3 Provided is a summary table listing the following information for each gene and each brain tissue analyzed: gene names (triplicated genes in Ts65Dn are in red), mean expressions (ME) of Ts65Dn and euploid mice, standard errors of MEs, CVs of the Ts65Dn and euploid samples, technical and biologic variance, mean trisomic:euploid gene expression ratios from electronic and RNA pools, and *P *values from *t*-test, *tu*-test, Wilcoxon test, permutation test, and F-test. Click here for file

Additional data file 4 Provided is a scatter plot of CVs of euploid versus Ts65Dn mice in brain tissues. The dotted lines represent the ± 20% CV deviations from the ideal correlation (plain line). Click here for file
