# Supplementary material for: Gene expression variation in Down's syndrome mice allows prioritization of candidate genes
Source: Genome Biol. 2007 May 25;8(5):R91. doi: 10.1186/gb-2007-8-5-r91 (PMC1929163; doi:10.1186/gb-2007-8-5-r91)
Supplement: Additional data file 1 — Provided is a table listing the references of the gene expression assay (Applied Biosystems) that were used for quantitative RT-PCR experiments. [file gb-2007-8-5-r91-S1.pdf]

Gene name	Assay ID	Amplicon size (bp)	GENE Reference NCBI
Nrip1	Mm00476537_s1	75	NM_173440,NM_173440,NM_008735,NM_008735
Usp25	Mm00450054_m1	90	NM_013918,NM_013918
Prss7	Mm00435990_m1	80	NM_178855,NM_178855,NM_008941,NM_008941
Ncam2	Mm00448056_m1	92	NM_010954,NM_010954
Mrpl39	Mm00489949_m1	127	NM_017404,NM_017404
Jam2	Mm00470197_m1	76	NM_023844,NM_023844
Gabpa	Mm00484598_m1	93	NM_008065,NM_008065
App	Mm00431827_m1	129	NM_007471,NM_007471
Adamts5	Mm00478620_m1	102	NM_011782,NM_011782
Usp16	Mm00470393_m1	60	NM_024258,NM_024258
Cct8	Mm00486840_m1	145	NM_009840,NM_009840
C21orf7	Mm00520021_m1	63	NM_144854,NM_144854
Cldn8	Mm00516972_s1	67	NM_018778,NM_018778
Tiam1	Mm00437071_m1	76	NM_009384,NM_009384
Ifnar2	Mm00494916_m1	57	NM_010509,NM_010509
Il10rb	Mm00434157_m1	68	NM_008349,NM_008349
Ifnar1	Mm00439544_m1	96	NM_010508,NM_010508
Ifngr2	Mm00492626_m1	107	NM_008338,NM_008338
Gart	Mm00599836_m1	83	NM_010256,NM_010256
Son	Mm00490912_m1	67	NM_019973,NM_019973
Itsn	Mm00495015_m1	72	NM_010587,NM_010587
Mrps6	Mm00459877_m1	82	NM_080456,NM_080456
Kcne2	Mm00506492_m1	75	NM_134110,NM_134110
C21orf51	Mm00507089_m1	85	NM_138743,NM_138743
Kcne1	Mm00434615_m1	67	NM_008424, XM_983408
Runx1	Mm00486762_m1	109	NM_009821,NM_009821
Cbr1	Mm00514232_m1	77	NM_007620,NM_007620
C21orf5	Mm00509870_m1	99	NM_026700,NM_026700
Hlcs	Mm00507322_m1	61	NM_139145,NM_139145
Dyrk1a	Mm00432934_m1	86	NM_007890,NM_007890
Ets2	Mm00468972_m1	66	NM_011809,NM_011809
Dscr2	Mm00517146_m1	80	NM_019537,NM_019537
Sh3bgr	Mm00489429_m1	121	NM_015825,NM_015825
B3galt5	Mm00473621_s1	78	NM_033149,NM_033149
Bace2	Mm00517133_m1	69	NM_019517,NM_019517
Fam3b	Mm00508056_m1	100	NM_020622,NM_020622
Znf295	Mm00558469_m1	69	NM_175428,NM_175428
Tff3	Mm00495590_m1	60	NM_011575,NM_011575
Tff2	Mm00447491_m1	69	NM_009363,NM_009363
Tmprss3	Mm00453694_m1	101	NM_080727,NM_080727
Pde9a	Mm00501039_m1	87	NM_008804,NM_008804
Wdr4	Mm00498663_m1	61	NM_021322,NM_021322
Cbs	Mm00460654_m1	124	NM_144855,NM_144855,NM_178224,NM_178224
Cryaa	Mm00660256_m1	74	NM_013501,NM_013501
Kiaa0179	Mm00551206_m1	80	NM_028244,NM_028244
Cstb	Mm00432769_m1	133	NM_007793,NM_007793
Col18a1	Mm00487131_m1	75	NM_009929,NM_009929
C21orf56	Mm00512974_m1	90	NM_029661,NM_029661
Lss	Mm00461312_m1	70	NM_146006,NM_146006
S100b	Mm00485897_m1	68	NM_009115,NM_009115
Hprt	Mm00446968_m1	64	NM_013556,NM_013556
Hmbs	Mm00660262_g1	111	NM_013551,NM_013551
