# Supplementary figures and images for: Gene expression variation in Down's syndrome mice allows prioritization of candidate genes
Source: Genome Biol. 2007 May 25;8(5):R91. doi: 10.1186/gb-2007-8-5-r91 (PMC1929163; doi:10.1186/gb-2007-8-5-r91)

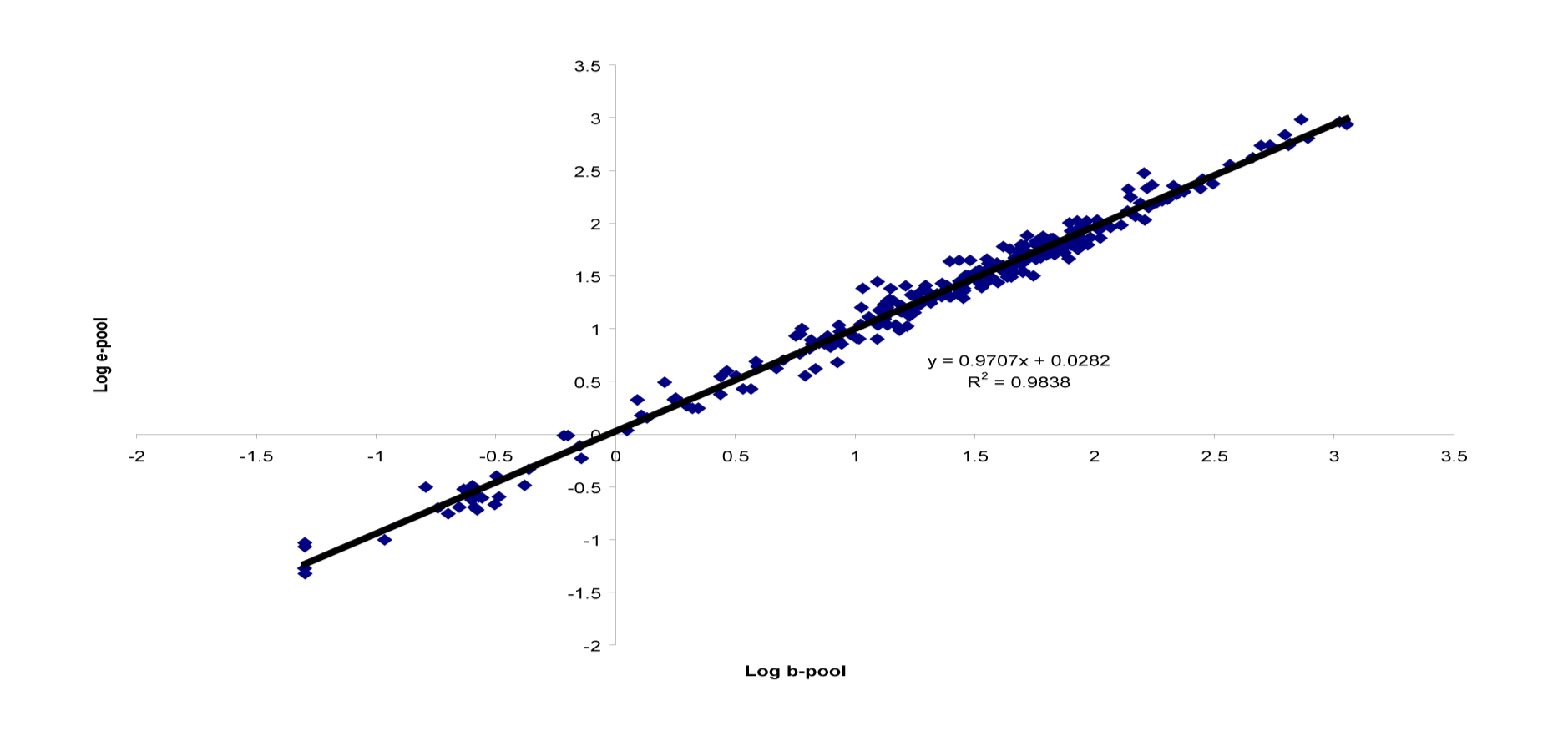

Supplement: Additional data file 2 — Provided is a figure of the correlation plot of intensities from electronic pools (y-axis) versus biologic pools (x-axis) for each gene in the three brain tissues. [file gb-2007-8-5-r91-S2.tiff]

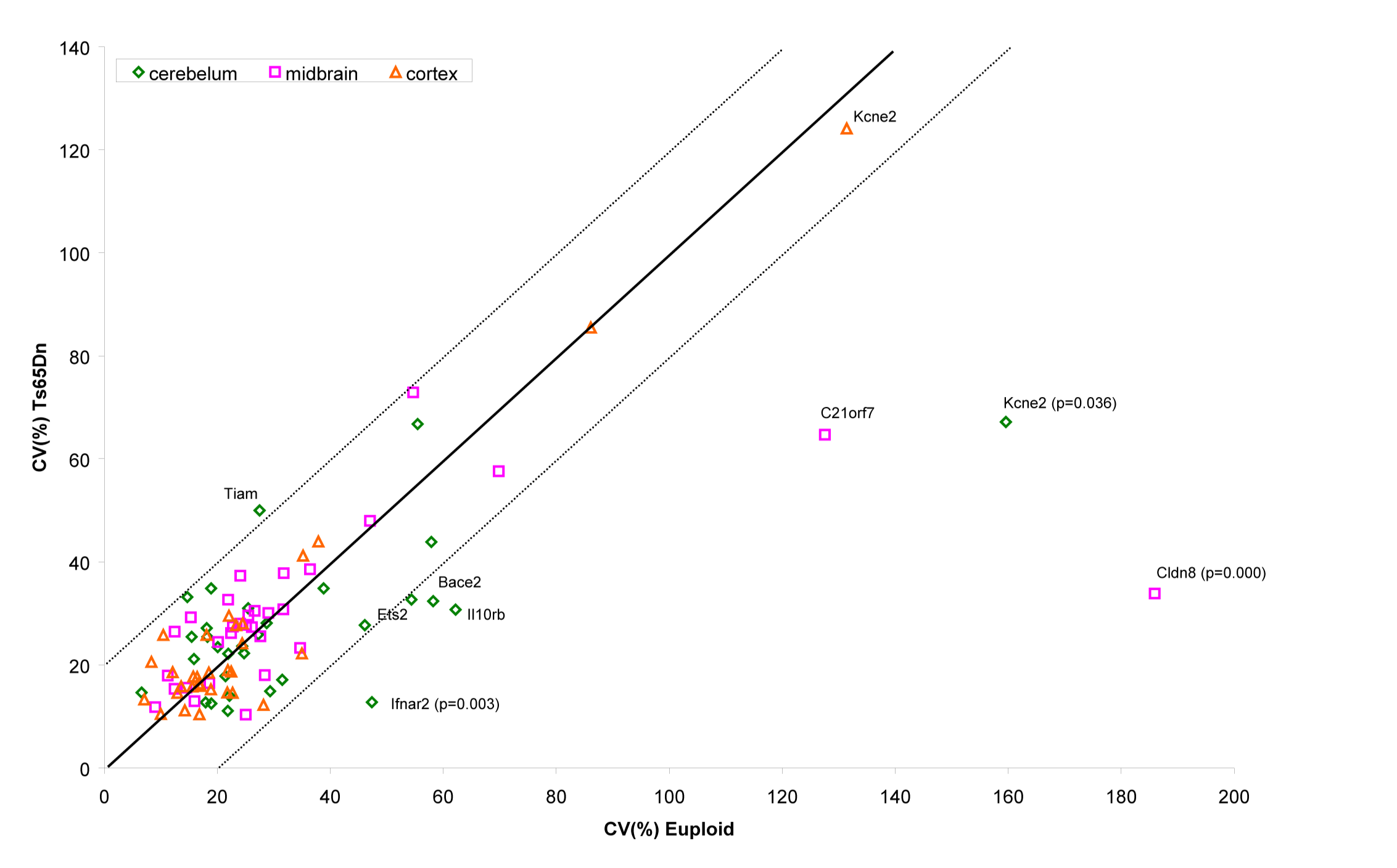

Supplement: Additional data file 4 — Provided is a scatter plot of CVs of euploid versus Ts65Dn mice in brain tissues. The dotted lines represent the ± 20% CV deviations from the ideal correlation (plain line). [file gb-2007-8-5-r91-S4.tiff]
