# Supplementary material for: Gene expression variation in Down's syndrome mice allows prioritization of candidate genes
Source: Genome Biol. 2007 May 25;8(5):R91. doi: 10.1186/gb-2007-8-5-r91 (PMC1929163; doi:10.1186/gb-2007-8-5-r91)
Supplement: Additional data file 3 — Provided is a summary table listing the following information for each gene and each brain tissue analyzed: gene names (triplicated genes in Ts65Dn are in red), mean expressions (ME) of Ts65Dn and euploid mice, standard errors of MEs, CVs of the Ts65Dn and euploid samples, technical and biologic variance, mean trisomic:euploid gene expression ratios from electronic and RNA pools, and P values from t-test, tu-test, Wilcoxon test, permutation test, and F-test. [file gb-2007-8-5-r91-S3.pdf]

	Gene Name	Mean Expression Ts65Dn	SE Ts65Dn	CV Ts65Dn	Technical Variance Ts65Dn	Biological Variance Ts65Dn	Mean Expression Euploid	SE Euploid	CV Euploid	Technical Variance Euploid	Biological Variance Euploid	Ts/Eu Ratio (e-pool)	Ts/Eu Ratio (b-pool)	P-value T-test	P-value Tu test	P-value Wilcoxon test	P-value Permutation test	P-valueF-test
Cerebellum	Nrip1	62,34	4,71	0,21	31,20	3722,29	63,40	8,14	0,36	68,12	11139,45	0,98	1,04	9,12E-01	9,13E-01	8,78E-01	8,01E-01	1,84E-01
	Usp25	60,05	4,15	0,20	118,96	2893,59	62,47	4,17	0,19	68,57	2923,26	0,96	1,07	6,87E-01	6,87E-01	7,21E-01	6,49E-01	9,30E-01
	Ncam2	56,49	9,11	0,46	9,06	13941,64	50,27	2,93	0,17	15,81	1445,59	1,12	1,25	5,26E-01	5,33E-01	1,00E+00	1,00E+00	1,56E-02
	Pde9a	75,06	8,98	0,34	225,92	13557,14	86,62	6,83	0,22	47,53	7839,31	0,87	0,58	3,23E-01	3,24E-01	1,95E-01	1,60E-01	2,93E-01
	Wdr4	8,74	0,76	0,25	1,45	97,56	8,51	0,65	0,21	1,48	70,11	1,03	1,25	8,27E-01	8,27E-01	1,00E+00	1,00E+00	7,23E-01
	Cbs	10,53	1,31	0,35	5,70	288,17	19,47	3,68	0,54	49,68	2280,90	0,54	0,58	3,84E-02	4,90E-02	4,66E-03	5,20E-03	2,90E-01
	Kiaa0179	8,00	0,54	0,19	5,91	49,84	8,14	0,74	0,26	3,25	91,17	0,98	1,03	8,85E-01	8,85E-01	7,98E-01	7,21E-01	4,69E-01
	Cstb	52,45	3,94	0,21	156,88	2601,47	57,09	4,58	0,23	38,31	3519,54	0,92	1,09	4,55E-01	4,55E-01	4,42E-01	3,81E-01	8,65E-01
	Col18a1	27,84	8,25	0,84	18,15	11424,63	43,53	23,23	1,51	34,24	90619,01	0,64	0,50	5,35E-01	5,41E-01	9,59E-01	8,74E-01	1,43E-01
	Lss	17,52	1,19	0,19	5,61	235,95	22,34	4,44	0,56	19,21	3309,03	0,78	1,03	3,12E-01	3,25E-01	6,45E-01	5,70E-01	1,09E-02
	S100b	545,13	93,40	0,48	4621,77	1465581,73	548,96	41,81	0,22	12254,04	293700,93	0,99	0,92	9,71E-01	9,71E-01	5,74E-01	5,04E-01	4,83E-02
	Mrpl39	72,05	5,68	0,22	30,12	5416,07	52,10	4,56	0,25	63,01	3487,00	1,38	1,62	1,59E-02	1,65E-02	3,79E-02	2,71E-02	7,90E-01
	Jam2	22,79	1,18	0,15	3,67	233,11	16,60	0,39	0,07	3,74	25,16	1,37	1,60	2,00E-04	8,96E-04	1,09E-03	9,00E-04	5,25E-02
	Gabpa	30,97	1,88	0,17	14,45	591,36	24,50	2,73	0,31	13,79	1250,32	1,26	1,33	7,10E-02	7,36E-02	1,05E-01	8,38E-02	1,31E-01
	App	864,92	39,02	0,13	19726,66	255796,34	573,15	36,30	0,18	11111,93	221344,38	1,51	1,72	8,18E-05	8,34E-05	1,55E-04	0,00E+00	3,91E-01
	Adamts5	1,76	0,22	0,35	0,34	7,86	1,43	0,20	0,39	0,45	6,49	1,23	1,64	2,71E-01	2,71E-01	3,28E-01	2,79E-01	7,74E-01
	Usp16	41,90	3,77	0,25	53,74	2389,19	31,85	1,74	0,15	19,43	507,03	1,32	1,24	2,97E-02	3,64E-02	4,99E-02	3,92E-02	2,10E-01
	Cct8	107,27	9,80	0,26	337,64	16150,72	69,97	6,74	0,27	85,34	7643,66	1,53	1,73	7,32E-03	8,31E-03	4,66E-03	3,00E-03	8,92E-01
	C21orf7	0,22	0,02	0,27	0,02	0,08	0,25	0,04	0,46	0,01	0,27	0,88	1,13	5,10E-01	5,14E-01	9,59E-01	8,81E-01	1,94E-01
	Cldn8	0,26	0,06	0,66	0,05	0,60	0,18	0,04	0,55	0,07	0,21	1,41	1,63	2,98E-01	3,02E-01	3,82E-01	3,24E-01	6,70E-01
	Tiam1	198,71	35,12	0,50	696,59	207213,61	141,35	13,74	0,27	257,98	31721,31	1,41	1,41	1,51E-01	1,62E-01	8,30E-02	6,61E-02	1,37E-01
	Ifnar2	61,84	2,80	0,13	143,75	1313,30	43,88	7,35	0,47	64,51	9081,02	1,41	1,56	3,86E-02	4,84E-02	1,04E-02	7,20E-03	2,65E-03
	Il10rb	20,14	2,19	0,31	16,95	808,17	14,82	3,26	0,62	12,90	1784,62	1,36	1,77	1,97E-01	2,00E-01	6,50E-02	4,42E-02	8,34E-02
	Ifnar1	70,87	5,92	0,24	45,67	5887,18	48,88	4,20	0,24	55,52	2970,88	1,45	1,60	9,03E-03	9,97E-03	4,66E-03	2,40E-03	9,40E-01
	Ifngr2	14,42	1,58	0,31	5,69	420,12	9,34	0,84	0,25	1,18	118,90	1,54	1,81	1,32E-02	1,67E-02	2,07E-02	1,47E-02	6,16E-01
	Gart	3,58	0,23	0,18	1,00	8,55	2,39	0,18	0,22	1,22	5,55	1,50	2,26	1,04E-03	1,14E-03	1,09E-03	2,00E-04	6,29E-01
	Son	548,36	45,44	0,23	6917,50	346934,50	418,22	29,69	0,20	2648,77	148104,32	1,31	1,41	3,10E-02	3,36E-02	3,79E-02	2,76E-02	6,93E-01
	Itsn	236,42	11,73	0,14	469,17	23126,51	168,78	13,19	0,22	834,41	29238,08	1,40	1,55	1,83E-03	1,88E-03	2,95E-03	1,80E-03	2,54E-01
	Mrps6	63,26	4,73	0,21	118,38	3759,24	39,97	2,24	0,16	49,86	840,94	1,58	1,58	5,48E-04	1,24E-03	1,09E-03	4,00E-04	4,63E-01
	Kcne2	34,86	8,28	0,67	6,92	11512,72	18,67	10,53	1,60	3,29	18621,71	1,87	2,44	2,47E-01	2,48E-01	2,34E-01	1,97E-01	3,62E-02
	C21orf51	59,39	5,35	0,25	145,89	4814,14	45,02	2,90	0,18	62,82	1416,94	1,32	1,50	3,34E-02	3,83E-02	2,07E-02	1,44E-02	3,97E-01
	Runx1	0,33	0,03	0,28	0,04	0,18	0,24	0,02	0,29	0,03	0,10	1,37	1,66	4,81E-02	4,96E-02	2,81E-02	1,94E-02	9,54E-01
	Cbr1	74,06	2,82	0,11	678,19	1335,68	54,83	4,24	0,22	48,94	3014,62	1,35	1,66	2,03E-03	2,56E-03	6,99E-03	4,60E-03	8,16E-02
	C21orf5	21,99	2,71	0,35	14,51	1231,22	14,23	0,95	0,19	13,70	152,57	1,54	1,54	1,72E-02	2,50E-02	2,81E-02	1,98E-02	1,30E-01
	Hlcs	84,19	4,43	0,15	146,87	3293,16	64,67	6,70	0,29	70,96	7547,20	1,30	0,99	2,91E-02	3,15E-02	4,99E-02	3,79E-02	9,40E-02
	Dyrk1a	213,10	16,71	0,22	540,41	46890,89	157,18	12,20	0,22	286,85	24982,91	1,36	1,52	1,71E-02	1,83E-02	8,30E-02	6,60E-02	9,79E-01
	Ets2	46,13	5,33	0,33	35,04	4780,58	34,40	6,57	0,54	116,61	7262,67	1,34	1,56	1,88E-01	1,88E-01	3,28E-01	2,78E-01	2,08E-01
	Dscr2	34,42	1,52	0,13	7,86	389,25	28,24	1,88	0,19	110,04	591,09	1,22	1,53	2,28E-02	2,34E-02	1,48E-02	9,60E-03	3,05E-01
	Sh3bgr	31,66	4,91	0,44	65,18	4056,11	27,46	5,62	0,58	58,73	5302,95	1,15	1,41	5,82E-01	5,82E-01	4,42E-01	3,85E-01	4,83E-01
	B3galt5	73,01	8,56	0,33	57,67	12321,64	45,62	2,37	0,15	53,81	940,57	1,60	1,69	8,12E-03	1,49E-02	4,66E-03	2,80E-03	4,69E-02
	Bace2	4,19	0,48	0,32	0,83	38,65	3,51	0,72	0,58	0,83	87,70	1,19	1,71	4,45E-01	4,47E-01	2,79E-01	2,31E-01	1,44E-01
	Znf295	20,09	1,93	0,27	9,87	625,18	16,69	1,07	0,18	9,03	192,75	1,20	1,38	1,46E-01	1,52E-01	1,95E-01	1,59E-01	3,09E-01
Midbrain	Nrip1	45,65	4,04	0,25	24,68	2739,77	47,37	3,46	0,21	42,40	2012,86	0,96	0,76	7,51E-01	7,51E-01	5,74E-01	5,07E-01	6,27E-01
	Usp25	44,59	3,44	0,22	70,49	1990,09	48,56	3,25	0,19	135,71	1776,08	0,92	0,52	4,16E-01	4,16E-01	1,95E-01	1,56E-01	7,17E-01
	Ncam2	100,66	8,04	0,23	234,45	10871,59	99,21	7,18	0,20	302,90	8662,21	1,01	0,75	8,95E-01	8,95E-01	8,78E-01	8,03E-01	8,00E-01
	Pde9a	25,52	2,29	0,25	20,96	879,89	25,63	1,97	0,22	20,41	652,40	1,00	0,83	9,70E-01	9,70E-01	8,78E-01	8,06E-01	6,94E-01
	Wdr4	8,87	0,95	0,30	4,14	151,10	8,50	0,80	0,27	2,75	107,99	1,04	1,04	7,71E-01	7,71E-01	1,00E+00	1,00E+00	7,49E-01
	Cbs	6,68	0,84	0,36	2,74	118,17	10,83	1,36	0,35	1,26	309,70	0,62	0,58	2,08E-02	2,35E-02	3,79E-02	2,55E-02	9,96E-01
	Kiaa0179	7,82	0,54	0,20	1,42	48,92	7,62	0,47	0,17	1,16	36,73	1,03	0,91	7,87E-01	7,88E-01	8,78E-01	8,02E-01	7,63E-01
	Cstb	39,65	1,25	0,09	111,47	260,41	40,26	2,03	0,14	113,64	694,69	0,98	0,94	8,02E-01	8,03E-01	6,45E-01	5,77E-01	2,33E-01
	Col18a1	2,12	0,38	0,50	1,11	23,97	3,11	0,62	0,56	1,54	64,09	0,68	0,77	1,94E-01	1,99E-01	2,34E-01	1,97E-01	7,83E-01
	Lss	18,95	2,69	0,40	5,79	1215,59	18,38	2,16	0,33	8,49	785,41	1,03	0,96	8,71E-01	8,71E-01	7,98E-01	7,20E-01	6,33E-01
	S100b	210,25	25,89	0,35	3199,00	112579,46	214,22	17,34	0,23	1023,39	50536,25	0,98	0,83	9,00E-01	9,01E-01	7,21E-01	6,49E-01	2,91E-01
	Mrpl39	62,52	3,41	0,15	263,54	1959,18	39,86	1,75	0,12	49,87	516,46	1,57	1,37	3,84E-05	1,26E-04	1,55E-04	0,00E+00	5,82E-01
	Jam2	14,92	1,61	0,30	6,11	432,98	11,00	1,03	0,27	5,42	178,98	1,36	1,20	5,89E-02	6,23E-02	4,99E-02	3,77E-02	7,28E-01
	Gabpa	24,14	3,29	0,39	27,28	1822,09	16,64	2,14	0,36	6,09	771,09	1,45	0,69	7,72E-02	8,06E-02	3,79E-02	2,63E-02	8,82E-01
	App	959,31	40,28	0,12	41709,59	272547,85	691,45	22,17	0,09	27488,18	82552,99	1,39	1,17	4,40E-05	1,20E-04	1,09E-03	2,00E-04	4,93E-01
	Adamts5	2,20	0,37	0,48	0,16	23,27	1,51	0,25	0,47	0,12	10,60	1,46	1,39	1,47E-01	1,50E-01	1,05E-01	8,09E-02	9,57E-01
	Usp16	24,05	0,88	0,10	12,13	129,52	18,25	1,62	0,25	5,52	438,96	1,32	1,03	7,05E-03	9,39E-03	3,79E-02	2,61E-02	3,24E-02
	Cct8	105,15	11,46	0,31	533,58	22079,89	65,12	7,31	0,32	150,94	8970,81	1,61	1,39	1,07E-02	1,24E-02	2,07E-02	1,47E-02	9,42E-01
	C21orf7	0,32	0,07	0,64	0,03	0,87	0,47	0,21	1,27	0,03	7,37	0,68	0,37	5,07E-01	5,14E-01	7,98E-01	7,17E-01	9,39E-02
	Cldn8	0,33	0,04	0,34	0,05	0,25	0,60	0,39	1,84	0,35	25,27	0,55	0,35	4,99E-01	5,10E-01	1,05E-01	8,09E-02	2,10E-04
	Tiam1	67,03	5,51	0,23	174,32	5098,49	41,93	5,14	0,35	45,29	4439,99	1,60	1,46	4,94E-03	4,97E-03	2,07E-02	1,60E-02	3,13E-01
	Ifnar2	59,90	8,01	0,38	93,48	10772,26	35,98	4,04	0,32	31,01	2743,76	1,66	1,26	1,84E-02	2,30E-02	2,07E-02	1,50E-02	6,57E-01
	Il10rb	19,70	1,91	0,27	7,55	612,46	14,76	1,19	0,23	9,37	237,47	1,33	1,41	4,55E-02	4,91E-02	4,99E-02	3,99E-02	6,37E-01
	Ifnar1	61,48	6,08	0,28	87,55	6217,81	41,50	3,52	0,24	61,44	2086,13	1,48	1,42	1,31E-02	1,57E-02	1,48E-02	1,01E-02	6,96E-01
	Ifngr2	15,91	1,01	0,18	9,64	172,12	10,05	0,40	0,11	4,70	26,85	1,58	1,77	9,46E-05	4,15E-04	3,11E-04	2,00E-04	2,39E-01
	Gart	2,68	0,23	0,24	0,31	9,05	1,87	0,13	0,20	0,30	2,92	1,44	1,85	8,44E-03	1,07E-02	2,07E-02	1,42E-02	6,05E-01
	Son	299,18	30,91	0,29	1000,88	160531,05	226,08	12,27	0,15	236,48	25273,72	1,32	0,75	4,53E-02	5,50E-02	6,50E-02	4,95E-02	1,11E-01
	Itsn	230,39	22,23	0,27	2026,97	83035,39	155,52	14,39	0,26	584,57	34805,70	1,48	1,12	1,35E-02	1,53E-02	3,79E-02	2,69E-02	9,15E-01
	Mrps6	44,55	2,59	0,16	21,79	1128,91	32,03	2,11	0,19	10,34	746,74	1,39	1,04	2,16E-03	2,31E-03	4,66E-03	3,10E-03	7,53E-01
	Kcne2	6,45	1,66	0,73	1,90	464,05	5,03	0,97	0,55	1,04	158,55	1,28	1,29	4,73E-01	4,76E-01	6,45E-01	5,74E-01	4,65E-01
	C21orf51	36,53	2,01	0,16	22,35	680,74	27,85	1,44	0,15	9,29	346,01	1,31	1,41	3,45E-03	3,97E-03	1,48E-02	1,17E-02	8,64E-01
	Runx1	0,40	0,04	0,25	0,06	0,22	0,30	0,03	0,28	0,02	0,14	1,33	1,37	4,96E-02	5,03E-02	6,50E-02	4,54E-02	8,12E-01
	Cbr1	76,68	3,27	0,12	692,89	1797,96	56,88	3,19	0,16	134,51	1704,76	1,35	1,18	6,83E-04	6,84E-04	1,86E-03	1,20E-03	4,89E-01
	C21orf5	16,94	1,65	0,28	90,65	458,76	10,85	0,97	0,25	34,30	156,71	1,56	1,54	6,71E-03	8,54E-03	6,99E-03	4,60E-03	8,14E-01
	Hlcs	62,30	6,64	0,30	300,38	7405,96	40,03	4,12	0,29	60,60	2849,14	1,56	1,30	1,28E-02	1,50E-02	1,48E-02	8,30E-03	9,28E-01
	Dyrk1a	176,83	16,53	0,26	316,52	45906,06	130,37	5,75	0,12	89,89	5559,29	1,36	1,03	1,89E-02	2,71E-02	6,50E-02	5,02E-02	6,58E-02
	Ets2	38,40	2,45	0,18	60,25	1004,85	24,56	2,44	0,28	69,24	999,96	1,56	1,39	1,30E-03	1,30E-03	4,66E-03	2,30E-03	2,64E-01
	Dscr2	26,89	2,81	0,30	10,25	1330,94	20,90	1,88	0,25	7,09	595,51	1,29	1,34	9,84E-02	1,02E-01	4,99E-02	3,67E-02	7,02E-01
	Sh3bgr	9,63	1,96	0,58	5,13	645,73	7,99	1,97	0,70	3,66	652,55	1,21	1,24	5,63E-01	5,63E-01	2,34E-01	1,97E-01	6,24E-01
	B3galt5	104,74	9,71	0,26	503,27	15836,45	71,60	5,70	0,23	211,95	5453,60	1,46	1,38	1,07E-02	1,30E-02	1,48E-02	9,30E-03	6,97E-01
	Bace2	4,87	0,64	0,37	0,84	69,21	3,97	0,34	0,24	0,76	19,25	1,23	1,32	2,37E-01	2,43E-01	2,79E-01	2,32E-01	2,72E-01
	Znf295	15,88	1,83	0,33	8,42	564,68	12,89	1,00	0,22	4,82	167,95	1,23	1,19	1,74E-01	1,80E-01	1,61E-01	1,25E-01	3,16E-01
Cortex	Nrip1	57,93	2,99	0,15	65,35	1503,12	59,45	2,62	0,12	109,92	1156,62	0,97	0,98	7,09E-01	7,09E-01	5,74E-01	5,09E-01	6,89E-01
	Usp25	49,93	5,03	0,29	27,87	4252,88	55,36	5,85	0,30	30,22	5747,63	0,90	0,85	4,93E-01	4,93E-01	3,28E-01	2,81E-01	9,04E-01
	Ncam2	91,19	8,65	0,27	45,43	12575,44	95,84	10,18	0,30	40,27	17422,47	0,95	0,90	7,33E-01	7,33E-01	8,78E-01	8,04E-01	7,73E-01
	Pde9a	22,79	2,39	0,30	11,90	960,95	20,15	1,95	0,27	6,93	639,24	1,13	1,05	4,05E-01	4,06E-01	2,79E-01	2,34E-01	8,38E-01
	Wdr4	7,25	0,24	0,09	1,11	9,34	7,17	0,24	0,09	1,17	9,48	1,01	0,95	8,03E-01	8,03E-01	7,98E-01	7,21E-01	9,63E-01
	Cbs	7,17	1,09	0,43	2,18	200,20	10,84	1,42	0,37	5,64	340,89	0,66	0,59	6,00E-02	6,13E-02	3,79E-02	2,69E-02	7,08E-01
	Kiaa0179	7,19	0,42	0,17	4,38	29,77	7,36	0,34	0,13	2,71	19,19	0,98	0,86	7,48E-01	7,48E-01	5,74E-01	5,04E-01	5,33E-01
	Cstb	27,50	2,82	0,29	40,64	1331,41	28,14	2,68	0,27	11,20	1209,29	0,98	0,92	8,72E-01	8,72E-01	8,78E-01	7,98E-01	8,56E-01
	Col18a1	0,98	0,20	0,59	0,42	7,03	0,98	0,19	0,56	0,41	6,34	1,00	1,04	9,94E-01	9,94E-01	1,00E+00	1,00E+00	8,93E-01
	Lss	14,07	0,48	0,10	3,79	38,79	15,45	0,78	0,14	5,66	102,77	0,91	0,82	1,53E-01	1,58E-01	2,34E-01	1,94E-01	3,20E-01
	S100b	107,21	5,84	0,15	160,96	5728,48	93,22	2,42	0,07	209,83	981,65	1,15	0,99	4,40E-02	5,32E-02	3,79E-02	2,82E-02	6,87E-02
	Mrpl39	47,05	3,16	0,19	26,76	1672,43	30,91	2,39	0,22	18,82	957,94	1,52	1,38	1,13E-03	1,30E-03	1,86E-03	1,40E-03	7,18E-01
	Jam2	10,79	0,55	0,14	9,50	51,19	7,81	0,35	0,13	3,84	21,12	1,38	1,46	4,60E-04	6,85E-04	1,09E-03	5,00E-04	7,59E-01
	Gabpa	21,40	1,58	0,21	56,88	419,14	14,56	0,43	0,08	3,74	30,75	1,47	1,42	9,26E-04	3,06E-03	3,11E-04	3,00E-04	2,66E-02
	App	918,37	34,30	0,11	40664,33	197652,82	639,92	22,50	0,10	22803,22	85030,01	1,44	1,36	8,76E-06	1,87E-05	1,55E-04	0,00E+00	8,77E-01
	Adamts5	1,09	0,09	0,22	0,17	1,24	0,78	0,10	0,35	0,11	1,55	1,40	1,58	2,96E-02	2,98E-02	3,79E-02	2,74E-02	2,62E-01
	Usp16	34,60	2,27	0,19	31,51	867,87	25,67	1,10	0,12	12,57	202,03	1,35	1,32	3,27E-03	5,30E-03	2,95E-03	1,60E-03	2,78E-01
	Cct8	91,75	6,07	0,19	167,74	6186,72	55,14	4,38	0,22	119,71	3219,19	1,66	1,42	2,38E-04	3,12E-04	6,22E-04	5,00E-04	6,42E-01
	C21orf7	0,19	0,03	0,43	0,01	0,15	0,21	0,03	0,38	0,03	0,12	0,94	1,03	7,53E-01	7,53E-01	7,98E-01	7,23E-01	7,20E-01
	Cldn8	0,21	0,03	0,39	0,05	0,14	0,11	0,02	0,40	0,05	0,04	1,89	1,67	9,50E-03	1,22E-02	1,04E-02	6,10E-03	9,47E-01
	Tiam1	60,06	3,11	0,15	164,99	1627,47	40,95	3,15	0,22	49,29	1670,51	1,47	1,39	7,16E-04	7,17E-04	2,95E-03	2,50E-03	3,18E-01
	Ifnar2	54,63	5,05	0,26	479,68	4293,27	32,97	1,21	0,10	36,03	247,62	1,66	1,55	9,47E-04	3,30E-03	1,55E-04	0,00E+00	2,66E-02
	Il10rb	17,67	1,16	0,19	41,87	226,67	12,60	0,83	0,19	5,48	114,62	1,40	1,41	3,15E-03	3,65E-03	4,66E-03	3,60E-03	9,95E-01
	Ifnar1	49,75	2,56	0,15	78,15	1098,75	34,91	2,80	0,23	21,68	1321,00	1,42	1,37	1,57E-03	1,59E-03	6,22E-04	3,00E-04	2,62E-01
	Ifngr2	15,55	0,57	0,10	2,26	54,98	9,26	0,55	0,17	2,75	51,22	1,68	1,49	1,55E-06	1,56E-06	1,55E-04	0,00E+00	2,26E-01
	Gart	2,69	0,26	0,28	0,60	11,63	1,75	0,14	0,23	0,30	3,51	1,54	1,63	7,39E-03	9,72E-03	1,48E-02	1,07E-02	6,65E-01
	Son	358,17	32,71	0,26	1916,68	179797,65	262,19	16,76	0,18	1291,37	47189,48	1,37	1,30	2,05E-02	2,52E-02	4,99E-02	3,89E-02	3,67E-01
	Itsn	162,76	13,79	0,24	1226,51	31926,07	116,41	9,99	0,24	745,30	16757,80	1,40	1,29	1,65E-02	1,77E-02	1,48E-02	1,04E-02	9,74E-01
	Mrps6	37,27	1,75	0,13	10,79	515,38	24,08	0,60	0,07	23,14	60,29	1,55	1,45	5,14E-06	6,88E-05	1,55E-04	0,00E+00	1,16E-01
	Kcne2	5,76	2,53	1,24	1,05	1074,03	2,13	0,99	1,31	0,30	163,72	2,71	3,34	2,02E-01	2,13E-01	5,74E-01	5,03E-01	8,87E-01
	C21orf51	31,05	1,66	0,15	285,43	460,23	24,12	1,34	0,16	16,24	302,52	1,29	1,33	5,82E-03	6,10E-03	1,48E-02	9,40E-03	9,13E-01
	Runx1	0,25	0,01	0,15	0,03	0,03	0,20	0,01	0,18	0,01	0,03	1,25	1,21	1,75E-02	1,75E-02	2,07E-02	1,64E-02	5,92E-01
	Cbr1	71,31	2,75	0,11	308,32	1269,88	53,37	2,71	0,14	325,31	1237,61	1,34	1,33	3,79E-04	3,80E-04	1,09E-03	1,10E-03	4,82E-01
	C21orf5	20,11	1,12	0,16	45,47	209,69	13,19	0,73	0,16	29,08	90,20	1,52	1,49	1,40E-04	2,26E-04	6,22E-04	4,00E-04	9,99E-01
	Hlcs	46,36	2,56	0,16	212,85	1096,93	31,50	1,48	0,13	90,43	368,95	1,47	1,31	1,84E-04	3,60E-04	1,55E-04	0,00E+00	6,87E-01
	Dyrk1a	188,33	18,60	0,28	907,89	58117,79	143,74	12,42	0,24	501,88	25902,41	1,31	1,31	6,60E-02	6,90E-02	8,30E-02	6,83E-02	7,33E-01
	Ets2	39,69	1,72	0,12	64,17	494,41	26,40	2,61	0,28	57,19	1146,80	1,50	1,36	8,05E-04	1,11E-03	1,55E-04	0,00E+00	4,41E-02
	Dscr2	21,97	1,23	0,16	45,50	255,90	16,83	0,99	0,17	13,79	166,19	1,30	1,23	5,95E-03	6,26E-03	2,07E-02	1,36E-02	8,98E-01
	Sh3bgr	4,81	1,45	0,86	10,42	355,15	4,18	1,27	0,86	9,27	272,86	1,15	1,23	7,53E-01	7,53E-01	4,42E-01	3,87E-01	9,87E-01
	B3galt5	40,92	2,55	0,18	17,01	1094,68	27,58	1,64	0,17	155,73	451,08	1,48	1,34	6,10E-04	8,84E-04	4,66E-03	2,50E-03	9,01E-01
	Bace2	4,37	0,46	0,30	1,43	35,23	3,56	0,28	0,22	0,51	13,01	1,23	1,23	1,50E-01	1,55E-01	1,05E-01	8,49E-02	4,61E-01
	Znf295	14,27	0,81	0,16	9,53	109,98	12,24	0,74	0,17	3,40	91,14	1,17	1,22	8,46E-02	8,47E-02	4,99E-02	3,77E-02	8,79E-01
